# The new H_2_S-releasing compound ACS94 exerts protective effects through the modulation of thiol homoeostasis

**DOI:** 10.1080/14756366.2018.1509211

**Published:** 2018-09-03

**Authors:** Daniela Giustarini, Valerio Tazzari, Ivan Bassanini, Ranieri Rossi, Anna Sparatore

**Affiliations:** a Department of Life Sciences, University of Siena, Siena, Italy;; b Department of Pharmaceutical Sciences, Università degli Studi di Milano, Milan, Italy

**Keywords:** Dithiolethione, hydrogen sulphide, homocysteine, acetaminophen toxicity, N-acetylcysteine ethyl ester

## Abstract

The synthesis of a new dithiolethione-cysteine ethyl ester hybrid, ACS94, its metabolites, and its effect on GSH levels in rat tissues and on the concentration of circulating H_2_S is described. ACS94 rapidly enters the cells, where it is metabolised to cysteine and the dithiolethione moiety ACS48. Experiments performed through the oral administration of ACS94 to healthy rats showed that it is capable of increasing the GSH levels in most of the analysed organs and the concentration of circulating H_2_S. Although the increase in GSH concentration was similar to that obtained by ACS48 and N-acetylcysteine ethyl ester, the H_2_S increase was long-lasting and more evident with respect to the parent molecules. Moreover, a decrease of homocysteine in several rat organs and in plasma was noted. This effect may represent a potential therapeutic use of ACS94, as hyperhomocysteinaemia is considered a risk factor for cardiovascular diseases. Lastly, ACS94 was more efficient than N-acetylcysteine in protecting the liver and kidneys against acute acetaminophen toxicity.

## Graphical Abstract

**Figure:**
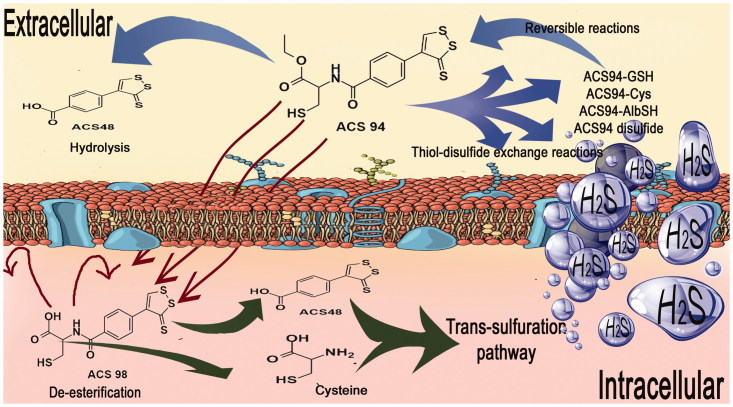

## Introduction

The physiological function of hydrogen sulphide (H_2_S) has recently been discovered, and a potential therapeutic use of this gas for the treatment of all diseases characterised by inflammation, oxidative stress, and glutathione (GSH) depletion has been suggested^1^ ^,^ ^2^. One possible approach for the therapeutic administration of H_2_S is represented by molecules capable of releasing it in a slow and controlled manner, thus mimicking that which takes place on a physiological level.

**Figure 1.**
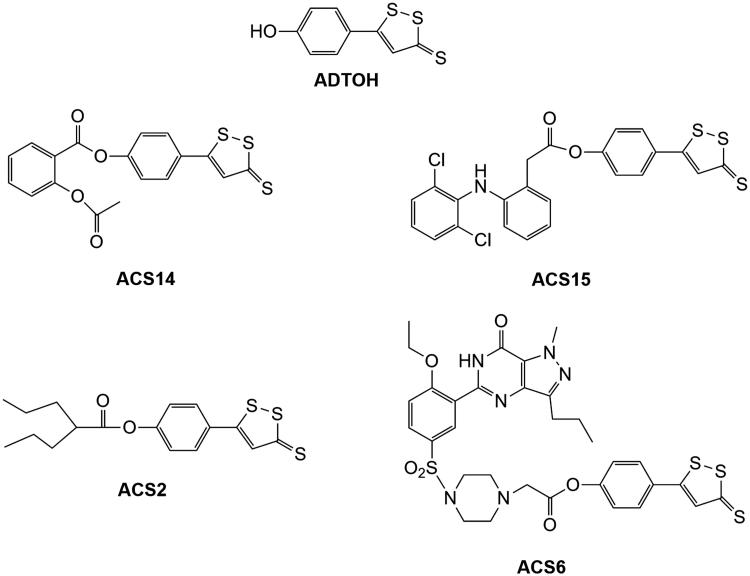
Chemical structure of several representative hybrids derived by conjugating ADTOH with existing drugs. ACS14: ADTOH grafted into aspirin; ACS15: ADTOH grafted into diclofenac; ACS2: ADTOH grafted into valproic acid; ACS6: ADTOH grafted into sildenafil.

We have experimented on two different classes of H_2_S-donating compounds: dithiolethiones and cysteine (Cys) prodrugs. Dithiolethiones are a class of sulphur-containing molecule, primarily of synthetic origin, but also found in trace amounts in certain cruciferous vegetables. They have been established as anticarcinogens and protective compounds against radiation injury and hepatotoxicity induced by carbon tetrachloride or acetaminophen (APAP)^3^. Dithiolethiones have been found to behave as H_2_S donors under physiological conditions^4^. Several hybrids were obtained by grafting 5-(4-hydroxyphenyl)-3*H*-1,2-dithiole-3-thione (ADTOH) into existing drugs^5^ ^,^ ^6^. The *in vitro* tests for these compounds suggest that, in addition to maintaining the parent molecule’s therapeutic capacities, they also have several other advantages (e.g. protection of gastric mucosa [ACS14, ACS15], much greater histone deacetylase inhibition than valproic acid [ACS2], blockage of superoxide formation, and downregulation of NADPH oxidase [ACS6]) (Figure 1)^7^. The common mechanisms through which the addition of the ADTOH moiety exerts these pharmacological effects seem to be based on the concomitant increase of both GSH and H_2_S, although several aspects still need to be clarified.

GSH plays an important role in the detoxification of xenobiotics, of their metabolites, and of reactive oxygen species (ROS), and would, therefore, be attractive for increasing its availability in man. As Cys concentration in cells is limiting to GSH synthesis, supplementing Cys or its precursors appears to be a logical choice for boosting its levels in cells. Cys is generally not used because it is rapidly oxidised to the insoluble cystine form, thus making the preparation of stable formulations difficult. Furthermore, it has been reported that Cys at high concentrations is toxic for cultured cells, and its content is therefore usually kept low in parenteral formulations^8^. This has stimulated the use of pharmacologically useful Cys prodrugs. The simplest derivative of cysteine is N-acetylcysteine (NAC), and numerous studies have already been reported in which NAC has been administered by either an oral or intravenous route^9^ ^,^ ^10^. In addition to the established pharmacological actions of NAC (mucolytic agent and antidote for acetaminophen intoxication), there are a plethora of others with the common effect of protecting against oxidative stress, a condition caused by an imbalance between pro-oxidant production and antioxidant defence, which is thought to be related to several pathological conditions, such as age-related diseases, atherosclerosis, diabetes, and cancer^11^. However, large clinical trials have failed to confirm this supposed protective effect of NAC^12^, thus stimulating the research of alternative molecules. We have recently studied N-acetylcysteine ethyl ester (NACET) as a new GSH enhancer. NACET is a derivative of NAC, and it is obtained through the esterification of its carboxylic group with ethanol. This modification renders the molecule more lipophilic, thus improving its pharmacokinetic behavior^13^. NACET resulted to be significantly more effective than NAC in increasing the circulating levels of both H_2_S and GSH content in several rat organs.

**Scheme 1.**
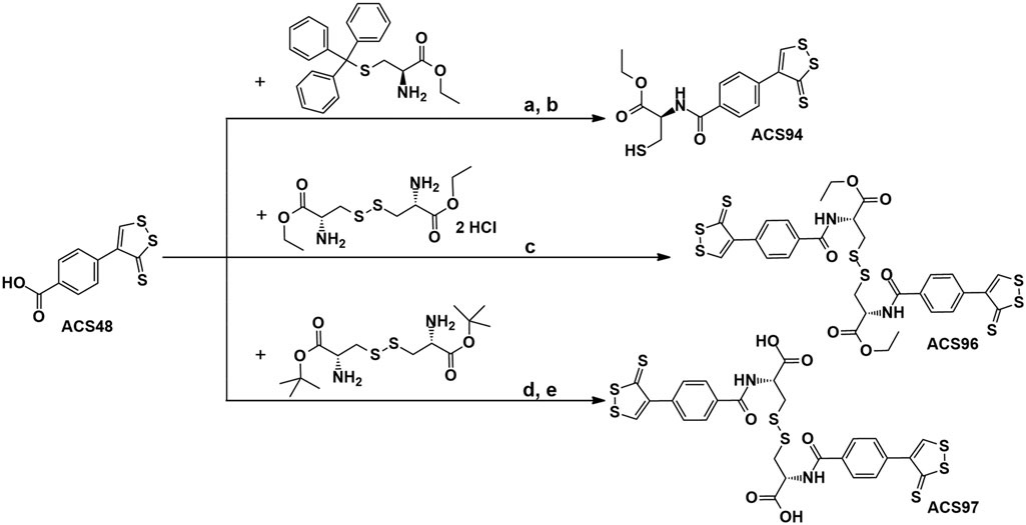
Reagents and conditions: (a) HOBt, EDAC, TEA, anh. DMF, 4h, r.t.; (b) 4N HCl in dioxane-H_2_O (90:10), 30 min, 0 °C; (c) HOBt, EDAC, DIPEA, anh. DMF, 24h, r.t.; (d) HOBt, EDAC, TEA, anh. DMF, 24h, r.t.; (e) 4N HCl in dioxane-H_2_O (86:14), 3h, r.t.

On these grounds, we have synthesised ACS94, a new dithiolethione-cysteine ethyl ester hybrid. ACS94 has been obtained by binding 4–(3-thioxo-3*H*-1,2-dithiol-4-yl)benzoic acid (ACS48) to the amino group of cysteine ethyl ester (Scheme 1), with the assumption that the two parts of the new chemical entity could have a synergic effect in increasing H_2_S levels, as well as in correcting the redox imbalance process present in several diseases, through an increase in tissue GSH. Here, the effect of ACS94 on the thiol pool and on H_2_S generation is evaluated in comparison to ACS48 and NAC/NACET. The protective effects against acetaminophen overdose are also investigated. Furthermore, the identification and the synthesis of the main metabolites of ACS94 are described.

## Experimental

### Materials and methods

#### General

Unless otherwise indicated, all the chemicals and solvents required for the syntheses and the analyses were of high purity grade and were purchased from Sigma-Aldrich (S.R.L., Milano, Italy). CC = flash column chromatography. Melting points (m.p.) were determined in open capillary tubes with a Büchi apparatus (Buchi Italia s.r.l., Cornaredo, Italy) and were uncorrected. ^1^H-NMR and ^13^C-NMR spectra were recorded with a Varian 300 MHz Oxford spectrometer (Varian, Palo Alto, CA) equipped with a non-reverse probe at 25° C, using DMSO-d_6_ as a solvent. The chemical shifts were expressed in ppm (*δ*), coupling constants (*J*) in Hertz (Hz). The high-resolution mass spectra (HRMS) were performed with an Apex II ICR-FTMS Bruker Daltonics mass spectrometer (Bruker Italia, Srl, Milano, Italy) in positive or negative electrospray ionization (ESI).

The *N*-acetylcysteine ethyl ester (NACET)^14^ and 4–(3-thioxo-3*H*-1,2-dithiol-4-yl)benzoic acid (ACS48)^15^ were synthesised according to the procedures used in the literature.

#### 3-Mercapto-2-(4-(3-thioxo-3H-1,2-dithiol-4-yl)benzamido)propanoic acid ethyl ester (ACS94)

A solution of (*S*)-trityl-L-cysteine ethyl ester^16^ (625 mg; 1.60 mmol) and trietylamine (TEA; 0.2 ml) in anhydrous N,N-dimethylformamide (DMF; 7 ml) was added to a solution of 4-(3-thioxo-3*H*-1,2-dithiol-4-yl)benzoic acid (ACS48; 407 mg; 1.60 mmol), 1-hydroxybenzotriazole (HOBt; 294 mg; 1.92 mmol), and 1-ethyl-3-(3-dimethylaminopropyl)carbodiimide hydrochloride (EDAC; 368 mg; 1.92 mmol) in anhydrous DMF (3 ml). The mixture was stirred for 4 h at r.t. under N_2_. Following the evaporation of the solvent under reduced pressure, the residue was taken up with CH_2_Cl_2_ and the organic phase was washed with water, dried over anhydrous Na_2_SO_4_, and evaporated to dryness. The crude compound was then purified by CC (silica gel; CH_2_Cl_2_ as eluent), yielding pure 2-(4-(3-thioxo-3*H*-1,2-dithiol-4-yl)benzamido)-3-(tritylthio)propanoic acid ethyl ester (659 mg; yield 66%). M.p. 86–93 °C. ^1^H-NMR (DMSO-d_6_): *δ* = 9.22 (s,1H); 8.96 (d, *J* = 7.91 Hz, 1H); 7.90 (d, *J* = 8.50 Hz, 2H); 7.68 (d, *J* = 8.50 Hz, 2H); 7.35–7.22 (m, 15H); 4.25–4.17 (m, 1H); 4.02–3.95 (q, *J* = 6.45 Hz, 2H); 3.31 (s, 1H); 2.83–2.71 (q, *J* = 12.90 Hz, 1H); 1.09–1.04 (t, *J* = 7.04 Hz, 3H).

The trityl deprotection of the thiol was performed by dissolving 2-(4-(3-thioxo-3*H*-1,2-dithiol-4-yl)benzamido)-3-(tritylthio)propanoic acid ethyl ester (240 mg; 0.38 mmol) in 4 N HCl in dioxane (10 ml) at 0 °C; water (1 ml) was added, and the mixture was stirred at 0 °C for 30 min. The solvent was evaporated to dryness, and the residue was taken up with ether and then evaporated. This procedure was repeated several times to ensure the complete elimination of HCl and dioxane. After rinsing the residue, first with CH_2_Cl_2_ and then with ether, pure ACS94 (117 mg; yield 80%) was obtained. M.p. 109–111 °C. ^1^H-NMR (DMSO-d_6_): *δ* = 9.22 (s, 1H); 8.84 (d, *J* = 7.63 Hz, 1H); 7.93 (d, *J* = 8.21 Hz, 2H); 7.68 (d, *J* = 8.21 Hz, 2H) 4.56–4.50 (m,1H); 4.11 (q, *J* = 7.03 Hz, 2H); 2.99–2.83 (m,1H); 2.68 (t, *J* = 7.92 Hz, 1H); 1.18 (t, *J* = 7.03 Hz, 3H). ^13^C-NMR (75 MHz, DMSO- d_6_): δ = 214.0, 170.8, 166.7, 160.4, 137.0, 133.0, 127.8, 129.3, 61.1, 56.1, 25.5, 14.5. HRMS (ESI) *m/z* calcd for C_15_H_15_NO_3_S_4_Na [M + Na]^+^: 407.98270; found: 407.98251.

#### Diethyl 3,3′-disulfanediylbis(2-(4-(3-thioxo-3H-1,2-dithiol-4-yl)benzamido)propanoate) ACS96

A solution of L-cystine ethyl ester dihydrochloride^17^ (300 mg; 0.81 mmol) and *N*,*N*-diisopropylethylamine (DIPEA; 0.68 ml; 3.9 mmol) in anhydrous DMF (3 ml) was added drop wise to a solution of ACS48 (413 mg; 1.9 mmol), HOBt (338 mg; 2.28 mmol), and EDAC (436 mg; 2.28 mmol) in anhydrous DMF (4 ml). The mixture was stirred at r.t. for 24 h, under N_2._ After the solvent was evaporated to dryness, the crude residue was taken up with CH_2_Cl_2_ and the organic solution was washed with water, dried over anhydrous Na_2_SO_4_, and evaporated to dryness. The crude compound was then purified by CC (silica; CH_2_Cl_2_/MeOH; 99:1). After rinsing with ether, pure ACS96 was obtained (222 mg; yield 36%). M.p. 98–102 °C.

^1^H-NMR: (DMSO-d_6_ *) δ* = 9.18 (s, 2H); 8.97 (d, *J* = 7.43 Hz, 2H); 7.87 (d, *J* = 8.26 Hz, 4H); 7.65 (d, *J* = 7.98 Hz, 4H); 4.77–4.70 (m, 2H); 4.16–4.09 (q, *J* = 7.15 Hz, 4H); 3.31–3.09 (m, 4H); 1.20–1.16 (t, *J* = 7.17 Hz, 6H). ^13^C-NMR (75 MHz, DMSO-d_6_): *δ* = 214.0, 171.0, 166.6, 160.4, 147.5, 137.0, 133.8, 129.3, 127.7, 61.5, 52.5, 14.5. HRMS (ESI) *m/z* calcd for C_30_H_28_N_2_O_6_S_8_Na [M + Na]^+^: 790.96052; found: 790.96138.

#### 3,3′-Disulfanediylbis(2-(4-(3-thioxo-3H-1,2-dithiol-4-yl)benzamido)propanoic acid) ACS97

A solution of cystine *tert*butyl ester^18^ (700 mg; 1.95 mmol) and trietylamine (TEA; 0.659 ml; 4.68 mmol) in anhydrous DMF (4 ml) was added to a solution of ACS48 (1g; 3.9 mmol), HOBt (716.5 mg; 4.68 mmol) and EDAC (896 mg; 4.68 mmol) in anhydrous DMF (4 ml) and the mixture was stirred at r.t. for 24 h, under N_2_. After the solvent was evaporated to dryness, the crude residue was taken up with CH_2_Cl_2_, and the organic solution was washed with water, dried over anhydrous Na_2_SO_4_, and evaporated to dryness. The crude compound was then purified by CC (silica gel; CH_2_Cl_2_/MeOH; 99:1). After rinsing with ether, pure *tert*butyl 3,3′-disulfanediylbis(2-(4-(3-thioxo-3*H*-1,2-dithiol-4-yl)benzamido)propanoate) was obtained (910 mg; yield 57%). M.p. 88–90 °C. ^1^H-NMR (DMSO-d_6_): *δ* = 9.18 (s, 2H); 8.97 (d, *J* = 7.43 Hz, 2H); 7.87 (d, *J* = 8.26 Hz, 4H); 7.65 (d, *J* = 7.98, 4H); 4.77–4.70 (m, 2H); 3.31–3.09 (m, 4H); 1.48 (s, 18H).

Water (5 ml) was added to a solution of *tert*butyl 3,3′-disulfanediylbis(2-(4-(3-thioxo-3*H*-1,2-dithiol-4-yl)benzamido)propanoate) (910 mg; 1.1 mmol) in 4 N HCl in dioxane (30 ml) at 0 °C as a scavenger, and the mixture was stirred at r.t. for 3 h. The solvent was evaporated to dryness and the residue was taken up with ether and then evaporated. This procedure was repeated several times to ensure the complete elimination of HCl and dioxane. After rinsing the residue, first with CH_2_Cl_2_ and then with ether, pure ACS97 (686 mg; yield 87%) was obtained. M.p. 254–255 °C. ^1^H-NMR (DMSO-d_6_): *δ* = 9.19 (s, 2H); 8.85 (d, *J* = 7.98 Hz, 2H); 7.87 (d, *J* = 8.25 Hz, 4H); 7.64 (d, *J* = 8.25 Hz, 4H); 4.73–4.67 (m, 2H); 3.55–3.07 (m, 4H). ^13^C-NMR (75 MHz, DMSO-d_6_): *δ* = 214.0, 172.4, 166.5, 160.2, 147.5, 136.9, 134.0, 129.2, 127.6, 52.5. HRMS (ESI) *m/z* calcd for C_26_H_19_N_2_O_6_S_8_ [M–H]^–^: 710.90143; found: 710.90386.

### Animals

Sprague–Dawley male rats (300 g) were purchased from Charles River (Calco, Milan, Italy). The rats were kept under controlled conditions (22–24 °C, relative humidity 40–50%, under a 12-h light/dark cycle) and fed *ad libitum* for 2–3 weeks before their use and during the experiments. All animal handling procedures were carried out in accordance with the European Community guidelines for the use of laboratory animals. The experiments were authorised by the local ethical committee of the University of Siena.

### Identification of the main metabolites of ACS94

The rats were implanted with either a single or double valve (model 415, 18 × 18 mm and model 620, 20 × 20 mm; Danuso Instruments, Milan, Italy). The jugular and femoral veins were cannulated (Dow Corning Silastic 0.51 mm id, 0.94 mm od) for compound administration (femoral) and blood collection (jugular), as previously described^19^. The valve was implanted under pentobarbital anaesthesia (50 mg/kg) 2 days prior to the experiment, and the animals were allowed to move about freely before and during the experiments.

The rats received intravenous (iv) administrations of ACS94 (20 mg/kg) through the femoral vein. At the indicated times, blood aliquots (200 μl each) were collected through the valve connected to the jugular vein in tubes containing 5 μl 50 mg/ml K_3_EDTA, and were immediately centrifuged at 10,000×*g* for 20 s to obtain plasma.

The analysis of the ACS94 metabolites in the plasma was carried out by deproteinising one aliquot of plasma (50 μl) through the addition of three volumes of acetonitrile. The supernatants were then acidified through the addition of trifluoroacetic acid and were loaded into HPLC. The HPLC separation was performed on a C18 column (Zorbax Eclipse XDB-C18 4.6 mm ×150 mm, 5 μm; Agilent Technologies, Milan, Italy). Elution conditions: solvent A = sodium acetate 0.25% (*v*/*v*) pH 3.09; solvent B = acetonitrile; 0–5 min: 94% solvent A/6% solvent B; 5–10 min linear gradient from 6% to 10% solvent B, 10–10.5 min linear gradient from 10% to 14% solvent B, 10.5–14.5 min 14% solvent B, 14.5–15 min linear gradient from 14% to 25% solvent B, 15–19 min linear gradient from 25% to 33% solvent B. A constant flow rate of 1.2 ml/min was applied. Detection was performed at 390 nm excitation and at 480 nm wavelength emission. Asymmetrical disulphide with Cys (ACS94-Cys) was produced by reacting 0.2 M ACS94 with 0.1 M cystine in a 10 mM phosphate buffer pH 8.0 for 2 days at room temperature. The identities of the peaks were analysed by adding the standard compounds, by analysing the same samples pretreated with dithiotreithol (DTT), and by comparing the retention times of the different peaks. One aliquot of plasma (20 μl) was used for H_2_S analysis.

For the study of drug distribution, animals were treated with endovenous infusion through the femoral vein with a solution of 20 mg/kg ACS94 or vehicle. After 60 min of the treatment rats were killed by decapitation, blood (about 2 ml) was collected in EDTA-containing tubes (50 μl of 50 mg/mL K_3_EDTA in saline) and organs were rapidly removed, washed in NaCl 0.9% (*w*/*v*) and then immediately frozen in liquid nitrogen and stored at –80 °C until analyses. Aliquots of blood (0.4 ml) were rapidly centrifuged at 10,000×*g* for 20 s to obtain plasma. Plasma was stored at –20 °C and used for the analysis of metabolites and total thiols. Erythrocytes were washed in Na^+^/K^+^ phosphate buffered saline containing 5 mM glucose and then haemolysed by the addition of 100 volumes of 0.02 M Na^+^/K^+^ phosphate buffer. One aliquot of frozen tissues was homogenised (1:10, *w*/*v*) by Teflon/glass potter in acetonitrile for the analysis of ACS94 and its metabolites (see procedure described above); one other aliquot was homogenised (1:5, *w*/*v*) in a 20% (*w*/*v*) TCA solution containing 1 mM K_3_EDTA and 0.2 mM diethylenetriaminepentaacetic acid (DTPA) for H_2_S detection. A third aliquot of frozen tissues was used for thiol analysis.

### Evaluation of GSH replenishing effect and modulation of Hcys levels

Animals implanted with a single valve for blood collection were treated orally (by gavage) with 10 mg/kg twice a day of ACS94 or equimolar NAC, NACET, ACS48 for four days. Aliquots of blood (0.2 ml) were collected every 12 h for H_2_S analysis. On the morning of the day after the last treatment (i.e. about 12 h from the last treatment), the rats’ blood and organs were rapidly removed under anaesthesia (pentobarbital, 50 mg/kg). Plasma was obtained through the centrifugation of whole blood at 10,000×*g* for 20 s. One aliquot (20 μl) was used for the fresh analysis of H_2_S, whereas the rest of the plasma was stored at –80 °C for the analysis of the total thiols. The organs were washed in NaCl 0.9% (*w*/*v*) and then immediately frozen in liquid nitrogen and stored at –80° until the time of analyses. The GSH was measured in the organ homogenates through HPLC after conjugation with monobromobimane (mBrB), as previously described^20^. After pretreatment with DTT and labelling with mBrB (see above), the total thiols (i.e. the sum of both the reduced and the disulphide form concentration) in the plasma was measured through HPLC.

### Production of hydrogen sulphide

H_2_S was detected through a modification of the methylene blue method^21^ coupled with HPLC detection, as previously described^22^. In short, 20 μl of fresh plasma was added to 40 μl of a 20% (*w*/*v*) TCA solution containing 1 mM K_3_EDTA and 0.2 mM DTPA. After 30 s of centrifugation at 10,000×*g*, 40 μl of the supernatant were added with 5 μl of N,N-dimethyl-*p*-phenylenediamine sulphate (DPD, 20 mM in 7.2 N HCl), followed by 5 μl of FeCl_3_ (30 mM in HCl 1.2 N); after 20′ of incubation in the dark, the samples were separated through HPLC using a Zorbax Eclipse XDB-C18 column (4.6 × 150 mm, 5 μm; Agilent Technologies). The methylene blue formed from the reaction of H_2_S with DPD in the presence of FeCl_3_ was monitored at a wavelength of 667 nm using a diode array detector.

### Protection against acetaminophen overdose

The animals were randomly divided into four groups, each consisting of four animals. APAP was dissolved in saline and injected ip at the dose of 2 g/kg. The group 1 rats served as a control group, and received a single dose ip injection of 1 ml saline containing 10% DMSO and 0.5% carboxymethylcellulose. The group 2 rats were treated with a single dose of APAP + vehicle. The group 3 and 4 rats received APAP + ACS94 (50 mg/kg) or equimolar NAC. The research drugs, dissolved in saline containing 10% DMSO and 0.5% carboxymethylcellulose, were administered orally 15 min before and 2 and 4 h after APAP administration. Six hours after the APAP treatment, the rats were anaesthetised with pentobarbital (60 mg/kg), after which blood from the abdominal aorta and organs were collected for biochemical analyses. After conjugation with mBrB, as above described, the GSH and Hcys were measured in the tissue homogenates through HPLC. After the pretreatment of the samples with DTT, the total Hcys was measured in the plasma through HPLC and mBrB labelling. The alanine aminotransferase, aspartate aminotransferase, lactate dehydrogenase, uric acid, urea, and creatinine in the plasma were measured using the Roche COBAS 6000 instrument (Roche Diagnostics S.p.A. Monza (MB), Italy). The protein SH groups (PSH) were determined in aliquots of TCA deproteinised samples: specifically, the protein pellets were re-suspended with a glass rod in a 0.2 M phosphate buffer, pH 7.4, containing 2% (*w*/*v*) sodium dodecyl sulphate, and were put in a shaker until complete dissolution. Aliquots of the samples were then diluted with the same buffer and reacted with an excess of DTNB, as previously described^23^.

An Agilent series 1100 HPLC (Agilent Tecnologies Italia, Cernusco sul Naviglio (MI), Italy). equipped with diode array and a fluorimetric detector was used for all determinations. All the spectrophotometric determinations were carried out using a Jasco V/550 instrument (Jasco Europe, Cremella (LC), Italy).

### Statistics

The data are expressed as means ± SD. The differences between the means were evaluated using one-way analysis of variance (ANOVA) with the Newman–Keuls multiple test, as appropriate. A value of *p* < .05 was considered statistically significant.

## Results

### Chemistry

3-Mercapto-2-(4-(3-thioxo-3H-1,2-dithiol-4-yl)benzamido)propanoic acid ethyl ester (ACS94) and its two main metabolites diethyl 3,3′-disulfanediylbis(2-(4-(3-thioxo-3H-1,2-dithiol-4-yl)benzamido)propanoate), ACS96 and 3,3′-disulfanediylbis(2-(4–(3-thioxo-3H-1,2-dithiol-4-yl)benzamido)propanoic acid) ACS97 have been synthesised, respectively, by coupling 4-(3-thioxo-3*H*-1,2-dithiol-4-yl)benzoic acid (ACS48)^15^ with (S)-trityl-L-cysteine ethyl ester^16^, followed by deprotection of the thiol group with HCl in dioxane, or with cystine ethyl ester dihydrochloride^17^, or with cystine *tert*butyl ester^18^ followed by deprotection of the carboxylic moieties with HCl in dioxane (Scheme 1). In all cases the reactions were performed at room temperature, in anhydrous DMF, using EDAC, HOBt, and TEA or DIPEA as coupling reagents.

### Biological results

#### ACS94 metabolism

**Figure 2.**
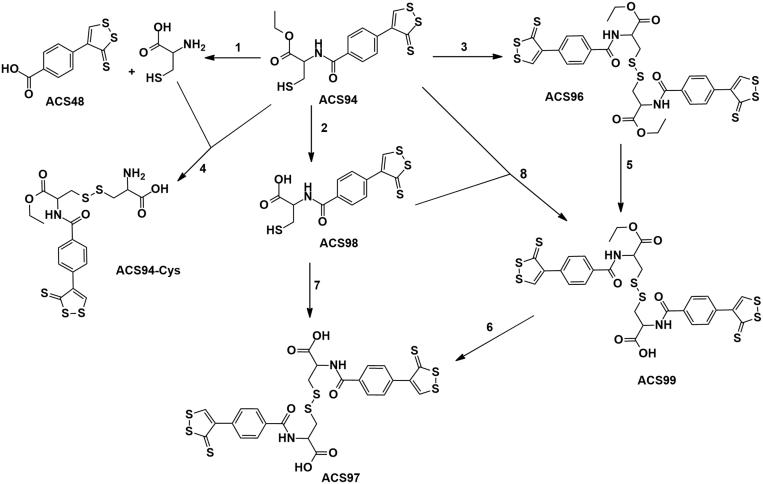
Scheme of the metabolic pathway of ACS94 leading to thiols (Cys and ACS98), symmetrical disulphides (ACS96, ACS97), asymmetrical disulphides (ACS94-Cys and ACS99).

**Figure 3.**
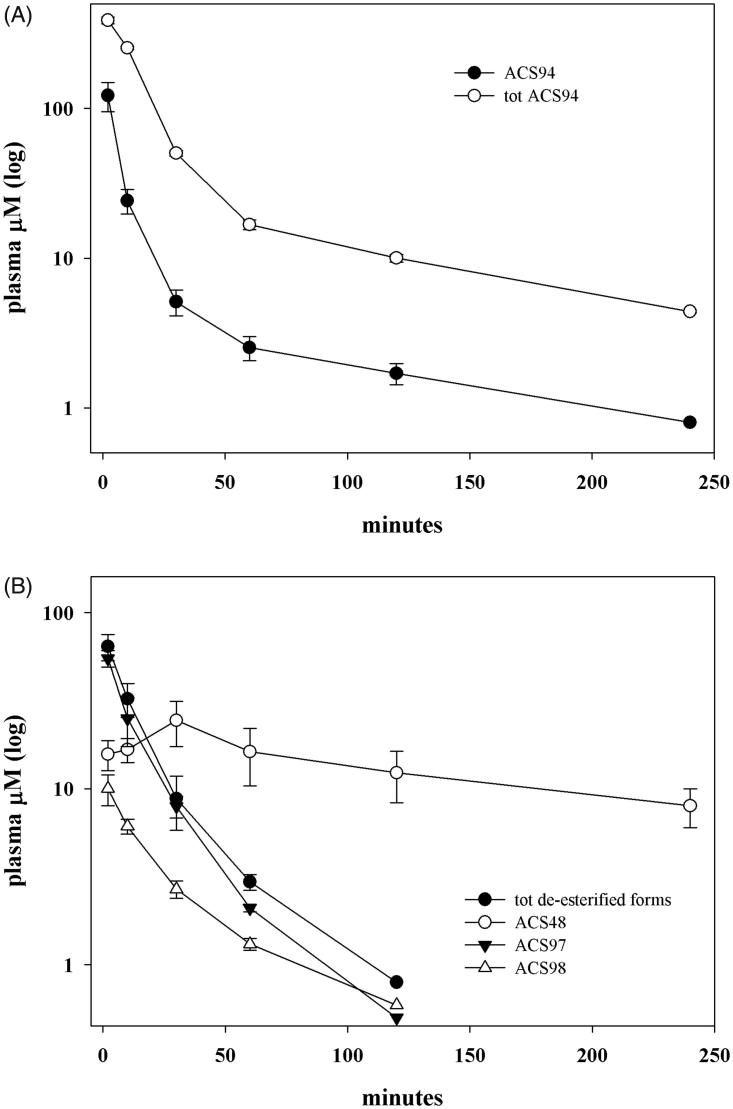
ACS94 metabolism. Plasma levels of ACS94 and its metabolites after the iv administration of 20 mg/kg to rats. Times of analysis are: 2′, 10′, 30′, 60, 120′, 240′. Data are the mean ± SD; *n* = 3. (A) tot ACS94= ACS94 + 2x ACS96 + ACS99 + 1/2 ACS94-Cys; (B) tot de-esterified forms: ACS98 + 2xACS97 + 1/2 ACS99.

The metabolism of ACS94 was firstly studied after its intravenous administration to rats. The metabolites investigated are reported in Figure 2. Specifically, ACS94 is supposed to undergo three different reactions: a) oxidation of the –SH group through thiol-disulphide exchange reactions; b) de-esterification by physiological esterases; c) hydrolysis of the amide bond between the parent compounds. As a result of these reactions, a lot of different metabolites may form. Firstly, it can produce both the parent molecules cysteine ethyl ester (CysEE) and then Cys and ACS48 (reaction 1), or it can be de-esterified to ACS98 (reaction 2). In addition, the –SH group can oxidise to form several symmetrical or asymmetrical disulphides: the symmetrical disulphides can form between either two ACS94 molecules (ACS96, reaction 3) or two de-esterified ACS94 molecules (ACS97, reaction 7); the asymmetrical disulphides may involve ACS94 with either Cys (reaction 4) or with ACS98 (to form ACS99, reaction 8), but, also, with all thiols and disulphides present in rat plasma. In addition, ACS99 can be obtained through the partial de-esterification of ACS96 (reaction 5), and can be hydrolysed to ACS97 (reaction 6). In Figure 3(A), the levels of ACS94 are reported in comparison with those of total ACS94. From this point onward, the term totACS94 is used to refer to all the metabolites of ACS94 that can be reversibly converted into this compound through the thiol-disulphide exchange reaction: ACS94 itself, the symmetric disulphide (i.e. ACS96), the ACS94-moiety in the asymmetrical disulphide (ACS99), and the ACS94-moiety in the asymmetrical disulphide with Cys. ACS94 disappeared rapidly from the plasma and was, in part, oxidised to ACS96 and ACS99. In fact, plasma is an oxidising compartment rich in disulphides^24^ that can oxidise ACS94 through several different thiol-disulphide exchange reactions (i.e. with the reactive –SH group of albumin and with low molecular mass thiols/disulphides). The concentration of the de-esterified metabolites (both in the reduced and oxidised form) and of the dithiolethione moiety (ACS48) is reported in Figure 3(B). All the compounds, with the exception of ACS48, exhibited near bi-exponential decay. The reason for these slight discrepancies with classical bi-exponential decay most likely lies in the thiol-disulphide exchange reactions that may occur and the great number of physiological thiols involved. Figure 3(A,B) suggests both a rapid metabolism and/or high distribution volume of ACS94, even if the latter is difficult to calculate accurately. It should be noted that ACS48 appeared immediately after iv infusion, and was still present at relevant concentrations after 4 h (Figure 3(B)).

NACET was shown to rapidly cross cell membranes and to then be de-esterified and trapped inside the cells^13^. To verify whether this mechanism also characterises ACS94 metabolism, a further experiment consisting in the iv administration of ACS94 and the analysis of the distribution of its metabolites in several rat tissues after 60 min from the treatment was carried out (Table 1). The data indicate that after 60 min from the treatment the major metabolites occurring in most of the analysed tissues (RBC included) are represented by the de-esterified forms of ACS94. The most abundant metabolite occurring intracellularly was always ACS98, the de-esterified form of ACS94, with a minor content of ACS48 and ACS94. The treatment also induced a significant increase in H_2_S in most of the studied tissues.

**Table 1.** Endovenous treatment with ACS94 and ACS48.

Treatment	ACS94	totACS94	tot de-esterified	ACS48	H_2_S
**Plasma**					
ACS94	2.53 ± 0.33	16.8 ± 2.5	2.96 ± 0.26	16.2 ± 0.6	0.355 ± 0.025^*^
Vehicle	–	–	–	–	0.251 ± 0.055
**RBCs**					
ACS94	0.984 ± 0.062	1.24 ± 0.023	14.6 ± 0.5	10.2 ± 0.3	ND
Vehicle	–	–	–	–	ND
**Liver**					
ACS94	2.23 ± 0.41	2.02 ± 0.54	20.1 ± 1.2	10.7 ± 0.9	0.407 ± 0.005^**^
Vehicle	–	–	–	–	0.222 ± 0.048
**Kidney**					
ACS94	0.847 ± 0.06	1.05 ± 0.08	18.4 ± 1.5	9.84 ± 0.7	0.369 ± 0.015^**^
Vehicle	–	–	–	–	0.185 ± 0.049
**Lung**					
ACS94	0.555 ± 0.031	0.784 ± 0.03	13.2 ± 1.8	7.25 ± 0.40	0.310 ± 0.061^*^
Vehicle	–	–	–	–	0.195 ± 0.010
**Heart**					
ACS94	0.355 ± 0.027	0.535 ± 0.089	8.45 ± 0.61	3.22 ± 0.028	0.286 ± 0.022
Vehicle	–	–	–	–	0.251 ± 0.113
**Brain**					
ACS94	0.236 ± 0.033	0.296 ± 0.060	8.56 ± 0.63	3.75 ± 0.13	0.449 ± 0.036^*^
Vehicle	–	–	–	–	0.294 ± 0.015

Metabolites of ACS94 in several rat tissues after a 60-min infusion with 20 mg/kg ACS94. Data are expressed as μM and are the mean ± SD of *n* = 4 treatments.

^*^ *p* > .05, ^**^ *p* < .01 vs vehicle.

#### Effect on GSH and homocysteine levels

**Figure 4.**
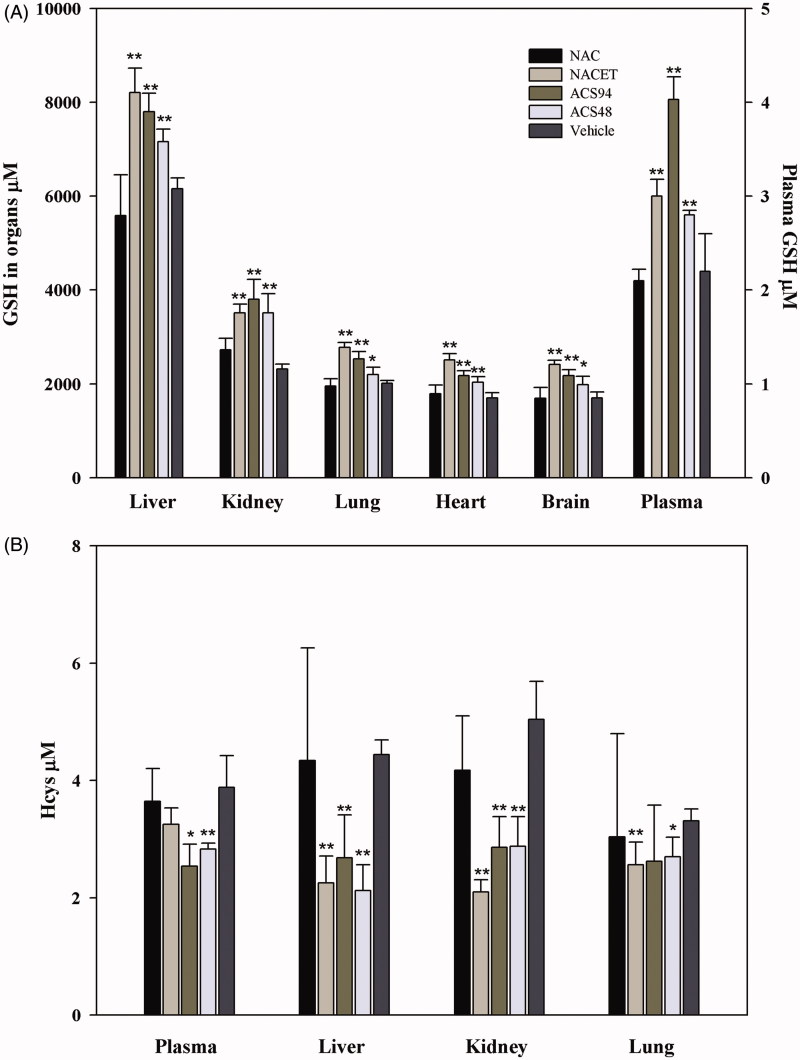
Concentration of GSH and Hcys in several rat organs and tissues after oral treatment. The rats were orally administered 10 mg/kg ACS94, equimolar ACS48, NAC, NACET or vehicle twice a day for 4 days. Glutathione (A) and homocysteine (B) were measured in organ homogenates through HPLC. The total GSH and total Hcys were measured in the plasma. Data are the mean ± SD; *n* = 3. **p < .001 vs vehicle; *p < .05 vs vehicle.

**Figure 5.**
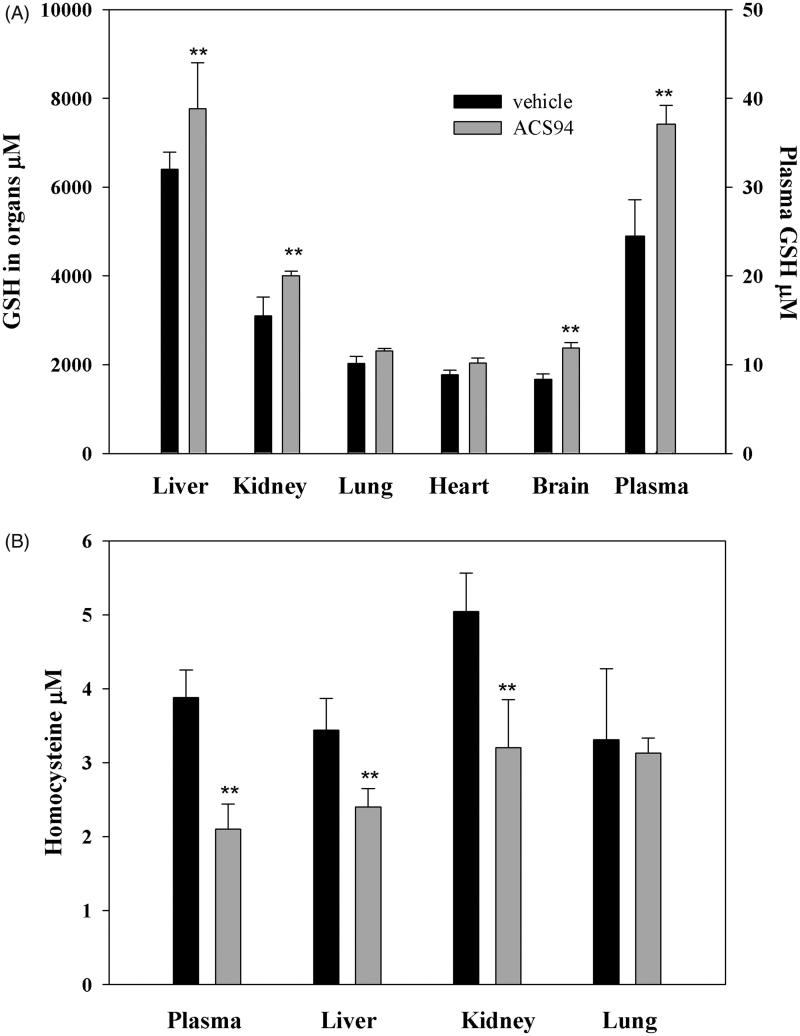
Concentration of GSH and Hcys in several rat organs and tissues after intravenous treatment. The rats were iv administered 20 mg/kg ACS94. After 60 min glutathione (A) and homocysteine (B) were measured in organ homogenates through HPLC. The total GSH and the total Hcys were measured in the plasma. Data are the mean ± SD; *n* = 3. **p < .001 vs vehicle.

To evaluate the ability of ACS94 to modulate the thiol to disulphide balance in blood and in solid tissues the drug was orally administered for 4 days in comparison with NAC, NACET, and ACS48. Our previous data indicate that by these selected conditions of treatment both NACET and some dithiolethione molecules are able to affect the levels of thiols^13^ ^,^ ^25^. The rats treated with ACS94 exhibited higher tissue GSH concentrations, particularly in the liver and kidneys. Significant increases in GSH vs both vehicle- and NAC-treated rats were also found in the lungs, heart, and brain. In addition, the treatment with ACS94 induced a significant rise of total GSH (i.e. the sum of the reduced and the oxidised forms of GSH) in plasma (Figure 4(A)). Conversely, the treatments were unable to influence tissue GSH levels in the other analysed tissues, namely the testis, spleen, and muscle (data not shown). This effect on GSH levels was similar to that induced by NACET and ACS48 (Figure 4(A)). ACS94 also increased GSH levels in some rat organs and in plasma after iv administration (20 mg/kg, Figure 5(A)). This effect was observed in the liver, kidneys, and brain.

Interestingly, the increase in GSH was in most cases associated to a decrease in Hcys. In the experiment where the drugs were orally administered for 4 days, ACS94 and ACS48 lead to a significant decrease of homocysteine in the plasma, kidneys, and liver. In the NACET-treated animals, a decrease of Hcys levels in the kidneys and liver was also observed, but not in the plasma (Figure 4(B)). This decrease was also found in the plasma, liver, and kidneys after single iv treatments with 20 mg/kg ACS94 (Figure 5(B)).

In addition to these data on thiol replenishment, we also studied the activity of some antioxidant enzymes in rat organs (namely glutathione reductase, glutathione peroxidase, superoxide dismutase) but we did not find any significant alteration induced by ACS94 (data not shown).

#### Hydrogen sulphide production

**Figure 6.**
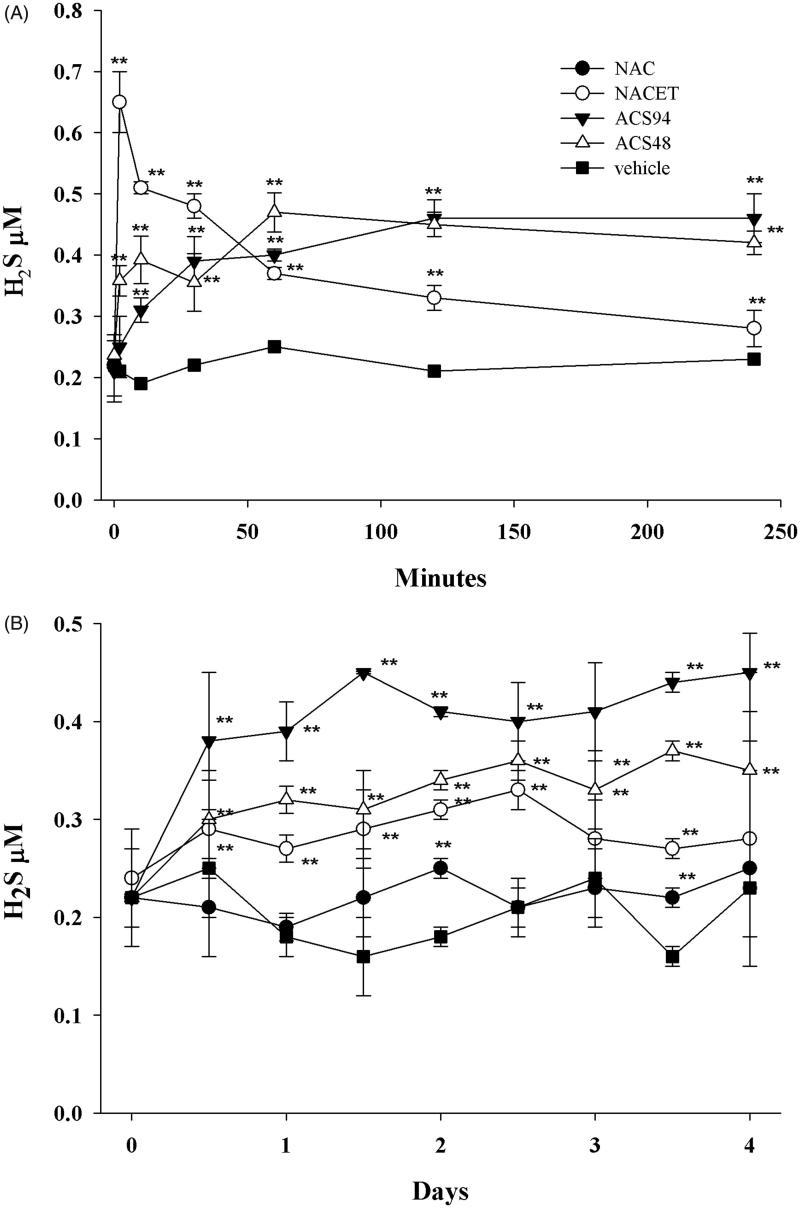
Concentration of H_2_S in plasma of rats treated with ACS94. The rats were either iv (A) or orally (B) administered with NAC, NACET, ACS94, ACS48, vehicle. For iv treatment, a dose of 20 mg/kg of ACS94 (or equimolar NACET and ACS48) was used. For oral treatment, 10 mg/kg of ACS94 (or equimolar NAC, NACET, ACS48) were administered twice a day for 4 days. The times of analysis for the iv treatment are: 2′, 10′, 30′, 60′, 120′, 240′. At the indicated times, the levels of H_2_S were measured in the plasma through colorimetric HPLC. Data are the mean ± SD; *n* = 3. **p < .001 vs vehicle.

H_2_S production from cells, *in vivo*, can be stimulated primarily by increasing cellular stores of cysteine, which can function as a substrate of cystathionine γ-lyase (CSE). We, therefore, compared the circulating levels of H_2_S after the iv or oral administration of ACS94 with those obtained after treatment with equimolar doses of NAC, NACET, or ACS48 (Figure 6(A,B)). As for the other analogous dithiolethione containing molecules, ACS94 increased the H_2_S levels in the rat plasma. The levels of H_2_S in the plasma from the iv treated rats peaked immediately after NACET infusion, whereas ACS94 evoked a significant but slower and more stable increase of H_2_S concentration (Figure 6(A)). ACS48 induced an increase of H_2_S levels with a pattern similar to ACS94. The H_2_S measured after the oral treatments indicated ACS94 as the best molecule for increasing its plasma levels (Figure 6(B)). No increase of H_2_S levels was observed in the rats treated with NAC.

#### Protection against acetaminophen toxicity

Acetaminophen metabolism is known to involve cytochrome P450 to form *N*-acetyl-*p*-benzoquinone imine (NAPQI), which causes hepatic damage. It has been suggested that NAPQI reacts with free thiol groups of proteins and, in turn, leads to mitochondrial damage and nuclear DNA fragmentation^26^. As NAPQI detoxification proceeds through GSH conjugation, GSH becomes depleted in APAP overdose. In addition to its ability to increase GSH production, as ACS94 bears an –SH group, it may also play a direct protective role against APAP damage. This potential protective effect in rats was studied in acute poisoning experiments, and in comparison with NAC, which is the drug clinically used to treat this kind of intoxication.

**Figure 7.**
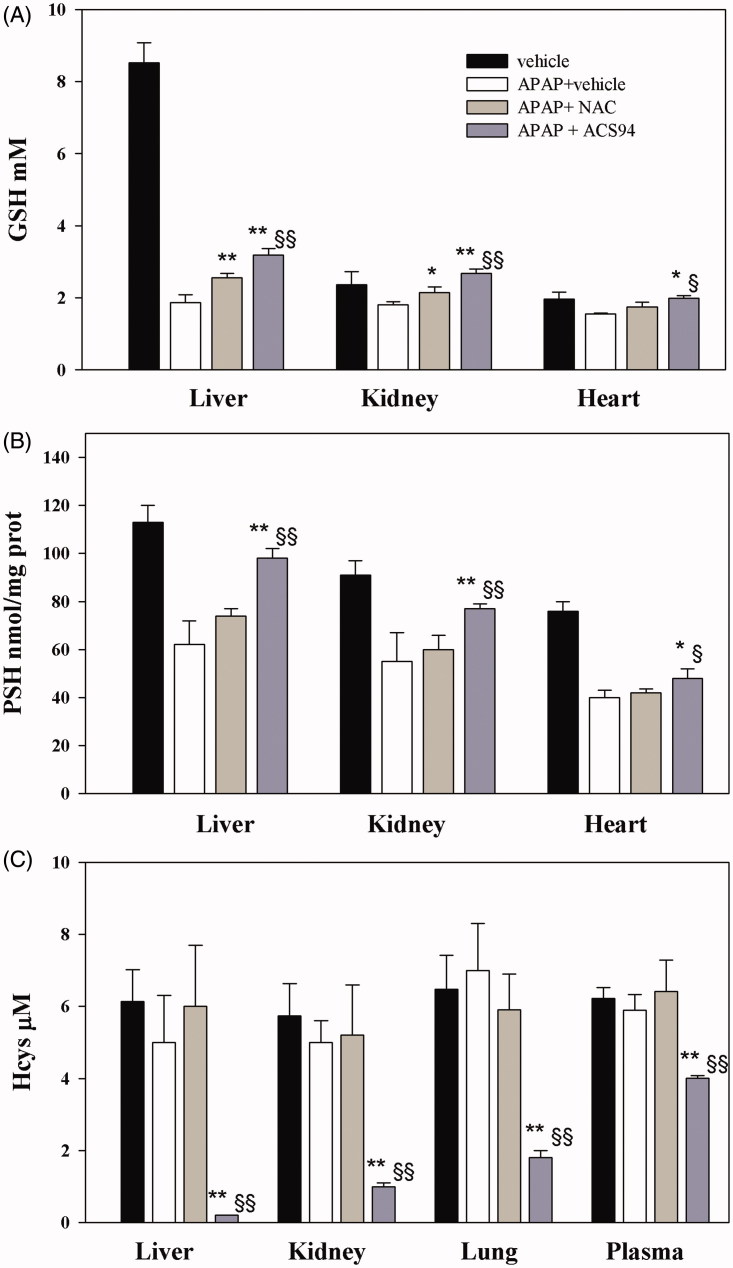
Protection against acetaminophen toxicity. Three out of four groups of rats were treated ip with 2 g/kg acetaminophen (APAP). 15′ before acetaminophen treatment and 2 h and 4 h after, 50 mg/kg ACS94 (or equimolar NAC) or vehicle was orally administered to rats. Vehicle group: rats treated with saline + DMSO and carboxymethylcellulose. The glutathione (GSH), protein thiols (PSH), and homocysteine (Hcys) were then measured in the organ homogenates through HPLC. The total Hcys was measured in the plasma. The GSH and PSH levels are reported for the liver, kidneys, and heart, whereas the homocysteine levels are reported for the liver, kidneys, and lungs. Data are the mean ± SD; *n* = 4 for each group *p < .05 vs APAP + vehicle; **p < .01 vs APAP + vehicle; ^§^ *p* < .05 vs APAP + NAC; ^§§^ *p* < .01 vs APAP + NAC.

Following the ip administration of 2 g/kg of APAP to the rats, a decrease of GSH and protein thiols was particularly evident in the liver but was also found in the kidneys and heart. The oral administration of ACS94 was more effective than NAC at preventing this decrease (Figure 7(A,B)) in all the studied organs (liver, kidneys, heart). The levels of Hcys were not influenced by the treatment with APAP but they decreased when the combined treatments APAP + ACS94 were carried out (Figure 7(C)). This effect was observed not only in the studied organs (liver, kidneys, lung), but also in the plasma, where the tHcys levels decreased significantly from 6.22 ± 0.3 to 4.01 ± 0.07 μM, *p* < .01). This decrease was not able to be observed in the NAC-treated animals. In addition, most of the indicators of liver/kidney damage were consistently lower in the ACS94 treated animals with respect to those treated with NAC (Table 2).

**Table 2.** Study of the protective effect of NAC and ACS94 on several damage biomarkers induced by paracetamol overdose in rats.

Sample	Urea (mg/dl)	Creatinine (mg/dl)	GOT/AST (U/L)	GPT/ALT (U/L)	LDH (U/L)	Uric acid (mg/dl)
Control	41.2 ± 2.0	0.37 ± 0.01	60.2 ± 9.23	62.2 ± 6.8	520 ± 12	0.30 ± 0.01
Paracetamol	43.1 ± 2.1	0.53 ± 0.02^##^	584 ± 11^##^	82.1 ± 3.35^#^	3229 ± 51^##^	0.66 ± 0.08^##^
Paracetamol + NAC	32.2 ± 3.2^**^	0.43 ± 0.58^*^	560 ± 20	78.0 ± 14.2	3003 ± 210	0.51 ± 0.02^*^
Paracetamol + ACS94	32.2 ± 4.7^**^	0.32 ± 0.02^**,§§^	322 ± 19^**,§§^	70.7 ± 6.4^**,§^	2006 ± 158^**,§§^	0.41 ± 0.05^**,§§^

Data are the mean ± SD. *n* = 4 for each group.

^*^ *p* < .05 vs paracetamol; ^**^ *p* < .01 vs paracetamol; ^§^ *p* < .05 vs NAC; ^§§^ *p* < .01 vs NAC; ^#^ *p* < .05 vs control; ^##^ *p* < .01 vs control.

## Discussion

This manuscript describes the basic features of several biochemical/pharmacological effects of the new chemical entity ACS94. ACS94 was synthesised with the aim of obtaining a new drug effective at increasing the levels of GSH and H_2_S, with the assumption that this could have beneficial effects on human health, mainly in the presence of pathologies characterised by redox imbalance, such as Parkinson disease^27^, Alzheimer’s disease^28^ ^,^ ^29^, osteoarthritis^30^, psoriasis, and skin diseases^31^.

GSH plays a protective role in relation to cellular oxidative damage, which mostly occurs during inflammation. If the oxidative stress is severe, glutathione disulphide (GSSG) may accumulate, leading to protein *S*-glutathionylation, with the activation/inactivation of several regulatory pathways^32^. A GSH decrease can also occur through irreversible conjugation with electrophilic species and the formation of chemically stable sulphides of GSH, which are further metabolised and excreted after the removal of glycine and glutamic acid and the acetylation of the cysteinyl amino group to form mercapturic acid^33^.

A decrease in GSH has been linked to the onset and/or progression of many diseases, and there is consequently considerable interest in counteracting this phenomenon. N-acetylcysteine has been widely studied as a GSH enhancer, as it is considered to be a source of Cys. However, despite the fact that it has been tested for treating numerous diseases, up until now it has only been used for paracetamol intoxication, since, in most cases, it did not work to ameliorate the symptoms^12^. Other compounds have therefore been studied as an alternative to NAC^8^ ^,^ ^34^.

Although H_2_S biology is quite complex, interest in it is growing. In fact, even if H_2_S is known to be toxic for humans by targeting cytochrome-c oxidase, there is much evidence regarding its protective role in several disease states, with these different effects being related to its levels. At low physiological concentrations, H_2_S can act as a modulator of numerous biochemical pathways, thus providing beneficial effects. Evidence of this is available for the nervous, gastrointestinal, cardiovascular, and respiratory systems^35^. Many studies have also been carried out with the aim of selecting compounds that can deliver H_2_S to target tissues. These essentially fall into three categories: sulphide salts, naturally occurring compounds, and synthetic H_2_S donors^5^. Although all of these seem to be promising, none of them have yet been accepted for targeted therapeutic applications. Therefore, research on this topic is ongoing.

We have recently studied two different synthetic compounds that provided promising results in experiments with rats. One of these is NACET, the ethyl ester of NAC. Chronic treatments with NACET in comparison with equimolar NAC indicated that only NACET was capable of increasing GSH significantly in most of the analysed organs. Furthermore, only NACET was able to cross the blood brain barrier, thus increasing the GSH in the brain. We found that the pharmacokinetic profile of this molecule is peculiar, in that it is readily absorbed and its bioavailability is quite high (more than 60%), whereas the bioavailability of NAC is below 5%. NACET was also able to increase the physiological levels of H_2_S in plasma^13^. Other Cys analogues have been shown to possess the ability to provide additional levels of H_2_S, such as S-propylcysteine, S-allylcysteine, S-propargylcysteine^36^. However, this property is not common to all Cys derivatives, since, under our experimental conditions, H_2_S levels did not increase in the NAC-treated animals.

Dithiolethiones were also shown to be effective at increasing GSH levels in several rat organs, including the brain, and H_2_S levels in plasma^15^ ^,^ ^22^ ^,^ ^25^ ^,^ ^37^. However, it is unclear how dithiolethiones are *per se* capable of increasing GSH levels. It has been hypothesised that several of the antioxidant enzymes involved in GSH synthesis/metabolism, such as γ-glutamylcysteine synthase, GSH transferase, and GSH reductase, may be altered by these drugs^3^ ^,^ ^38^. Similarly, although the mechanism by which dithiolethiones modulate H_2_S levels is still unknown, in our experiments we were able to observe a biphasic increase in the same, which can be explained by admitting the involvement of two different pathways.

ACS94 was formally obtained by conjugating a dithiolethione moiety (ACS48) with cysteine ethyl ester. As hypothesised, it maintained the ability to increase both GSH and H_2_S levels in rat organs and plasma, respectively (Figures 4(A), 5(A), and 6). ACS94 was as efficient as NACET and ACS48 at increasing GSH levels in rat organs: in fact, liver, kidneys, lung, and heart showed higher GSH levels with respect to the vehicle-treated animals. However, our data indicate that the concomitant release of Cys and a dithiolethione moiety does not increase GSH levels in a synergic way. This can be explained by the fact that γ-glutamylcysteine synthetase (GCL, that catalyses the first step of GSH synthesis) is regulated by non-allosteric feedback competitive inhibition (with glutamate) through GSH^39^. However, ACS94 was found to be the best H_2_S-releasing molecule in terms of both long lasting effect and H_2_S plasma levels reached (Figure 6(B)). The dithiolethione moiety seems to be responsible for this effect, as ACS48 and ACS94 behaved similarly, whereas the increase induced by NACET was less durable.

The metabolism of ACS94 is quite complex, as the molecule can undergo: de-esterification reactions, hydrolysis reactions, and thiol oxidation to disulphide by thiol-disulphide exchange reactions. As a consequence of this, many metabolites can form (Figures 2 and 3). The thiol-disulphide exchange reactions render the metabolism of ACS94 particularly complex, as these are reversible reactions and can involve many different thiol/disulphides physiologically present in biological fluids. It should be also noted that the disulphide metabolites of ACS94 can be distributed and metabolised in a different way with respect to the reduced metabolites. For example, a fraction of the disulphides can covalently bind to the Cys 34 of albumin, a rather peculiar kind of interaction for a drug, which makes its metabolism even more complex. As a matter of fact, the equilibrium between the bound fraction and the free drug is governed by the presence of low molecular mass thiols (LMM-SH) according to the reaction (1), and the concentration of these molecules changes greatly under several physio-pathological conditions, even depending on the different vascular districts^24^ ^,^ ^40^:

(1) PSH+LMM−SS↔PSS−LMM+LMM−SH.

However, after 60 min from the treatment with ACS94, we could observe a higher concentration of its de-esterified forms within cells of all the studied rat tissues with respect to the plasma. It is probable that, in analogy to NACET, the fraction of ACS94 that is de-esterified is trapped within cells, as it becomes more hydrophilic, and, consequently, its exit is restricted. Within the cells, it is then hydrolysed mainly to cysteine and the dithiolethione moiety. Both cysteine and ACS48 are efficient GSH enhancers (Figure 4). The presence of the ethyl ester group in the molecule, increasing its lipophilicity, likely favours the membrane crossing and trapping of ACS94 (after de-esterification) within cells, similarly to NACET. However, unlike NACET, the occurrence of the dithiolethione moiety allows for both a rapid increase and a longer maintenance of H_2_S production.

The evidence that ACS94 also maintained the ability to lower Hcys in plasma (and in some rat organs, namely liver, kidneys, and lung) (Figures 4(B) and 5(B)) may be of pharmacological interest. High levels of Hcys are suspected to contribute to the pathogenesis of cardiovascular diseases and other chronic conditions^41–43^. Until recently, the pharmacological treatments for hyperhomocysteinaemia have primarily focused upon the supplementation of folic acid and other B vitamins. However, while effective at decreasing Hcys levels, these vitamins do not seem to be capable of decreasing the incidence of cardiovascular events in humans^44^ ^,^ ^45^. Meanwhile, alternative approaches to reducing Hcys concentrations are being researched, and ACS94 may represent a promising drug for this purpose. We had already reported that other molecules containing dithiolethiones, such as ADTOH, ACS14, and ADT, lower the concentration of Hcys^36^ ^,^ ^37^. We can hypothesise that this Hcys-lowering effect may be related to the concomitant increase in GSH and H_2_S. In fact, the decrease in Hcys could result from the stimulation of the transsulphuration pathway (TSP), which is known to be redox regulated^46^. By influencing the thiol-disulphide balance, ACS94 can cause an enhancement of TSP, which, in turn, may be responsible for the increased Cys levels we measured in the rat organs (data not shown), as well as the increased GSH levels. In fact, the observed increase of H_2_S could be due to both enhanced TSP and increased Cys levels. This might also explain the biphasic release of H_2_S from the dithiolethiones: the initial increase (observed immediately after the drug’s administration) is likely due to its direct release from the dithiolethione-containing drugs, whereas the later increase (6–24 h after the treatments) may be attributable to the activation of TSP and generation from Cys. Moreover, our previous data indicate that dithiolethione treatments are followed by enhanced Cys and GSH release from the intracellular compartment to the blood, thus leading to an increase in the thiol to disulphide ratio in the plasma. Considering that Hcys mainly occurs in plasma as a disulphide (both with proteins and with low molecular mass thiols), the thiols occurring in the plasma can react with these disulphide forms to release free Hcys, which, in turn, can be metabolised. NACET was also able to decrease Hcys, and, like the Cys prodrug, we can hypothesise that it may promote a decrease in Hcys levels by reducing the disulphide forms of Hcys itself in the extracellular milieu, but without any involvement of the TSP.

Another pharmacological effect that ACS94 may exert is that of liver protection against intoxication with APAP. As a supplier of GSH, NAC is regarded as the antidote of choice for treating acetaminophen overdose. However, under our experimental conditions, NAC was only shown to exert some degree of protection against kidney toxicity (see Table 2, data of creatinine and uric acid), whereas ACS94 was more efficient at also protecting the liver against APAP damage, probably by virtue of the dithiolethione moiety, as well as its better pharmacokinetics with respect to NAC. In fact, only ACS94 was able to significantly increase the levels of GSH and protein thiols (PSH) in both the liver and kidneys (Figure 7). These data are consistent with previous observations, which indicated both NACET and dithiolethiones as hepatoprotective agents against APAP damage^13^ ^,^ ^47^.

One limit of this study stays on the lack of information about the toxic potential of the tested compound. The only information we have comes from the 4-day oral treatment (10 mg/kg twice a day). Along this time, we could not observe any change in the rat weight for every kind of administered drug (ACS94, ACS48, NAC, NACET). Further long-lasting experiments, with targeted experiments and dose–response studies are necessary to characterise the toxic potential of ACS94.

## Conclusions

In this article we have proposed a new synthetic compound that possesses several promising pharmacological properties: 1) it has good pharmacokinetics, as it rapidly enters the cells; 2) it releases H_2_S *in vivo*, and this effect is long-lasting; 3) it is capable of providing antioxidant protection by increasing GSH and by possessing an –SH group itself; 4) it significantly decreases Hcys levels in several rat target organs and in plasma; 5) it prevents APAP induced thiol depletion in rat kidneys and liver. ACS94 can affect the metabolic pathways relating to Hcys, Cys, GSH (by TSP pathway), and H_2_S. The dysregulation of these pathways is associated with several pathologies, mostly at a cardiovascular level^45^. By decreasing Hcys and increasing H_2_S and GSH, ACS94 is potentially capable of protect against these disorders. Moreover, thanks to its antioxidant properties, it can have beneficial effects by protecting the cells against ROS overproduction, which mostly occurs in inflammatory diseases. Considering that acetaminophen poisoning currently represents the most common cause of ALF in North America and Europe^48^, the protection offered against APAP intoxication is another effect worth of further investigation.
